# Tpl2 contributes to IL-1β-induced IL-8 expression via ERK1/2 activation in canine dermal fibroblasts

**DOI:** 10.1371/journal.pone.0259489

**Published:** 2021-11-04

**Authors:** Atsuto Naruke, Rei Nakano, Junichi Nunomura, Yoko Suwabe, Masumi Nakano, Shinichi Namba, Taku Kitanaka, Nanako Kitanaka, Hiroshi Sugiya, Tomohiro Nakayama

**Affiliations:** 1 Laboratories of Veterinary Radiotherapy, Nihon University College of Bioresource Sciences, Kameino, Fujisawa, Kanagawa, Japan; 2 Laboratory for Cellular Function Conversion Technology, RIKEN Center for Integrative Medical Sciences, Suehiro-cho, Tsurumi, Yokohama, Kanagawa, Japan; Chang Gung University, TAIWAN

## Abstract

In autoimmune diseases, fibroblasts produce and secrete various cytokines and act as sentinel immune cells during inflammatory states. However, the contribution of sentinel immune cells (i.e. dermal fibroblasts) in autoimmune diseases of the skin, such as atopic dermatitis, has been obscure. The pro-inflammatory cytokine interleukin 1β (IL-1β) induces the expression of chemokines, such as interleukin 8 (IL-8), in autoimmune diseases of the skin. IL-8 induces the activation and recruitment of innate immune cells such as neutrophils to the site of inflammation. IL-1β-mediated induction of IL-8 expression is important for the pathogenesis of autoimmune diseases; however, the intracellular singling remains to be understood. To elucidate the mechanism of the onset of autoimmune diseases, we established a model for IL-1β-induced dermatitis and investigated MAPK signaling pathways in IL-1β-induced IL-8 expression. We also identified that a MAP3K Tpl2 acts as an upstream modulator of IL-1β-induced ERK1/2 activation in dermal fibroblasts. We observed an increase in the expression of IL-8 mRNA and protein in cells treated with IL-1β. ERK1/2 inhibitors significantly reduced IL-1β-induced IL-8 expression, whereas the inhibitor for p38 MAPK or JNK had no effect. IL-1β induced ERK1/2 phosphorylation, which was attenuated in the presence of an ERK1/2 inhibitor. IL-1β failed to induce IL-8 expression in cells transfected with siRNA for ERK1, or ERK2. Notably, a Tpl2 inhibitor reduced IL-1β-induced IL-8 expression and ERK1/2 phosphorylation. We confirmed that the silencing of Tpl2 in siRNA-transfected fibroblasts prevented both in IL-1β-induced IL-8 expression and ERK1/2 phosphorylation. Taken together, our data indicate the importance of Tpl2 in the modulation of ERK1/2 signaling involved in the IL-1β-induced development of autoimmune diseases affecting the dermal tissue, such as atopic dermatitis.

## Introduction

Interleukin-8 (IL-8) is a member of the CXC chemokine family, and then is alternatively known as CXCL8. One of its roles is the activation and recruitment of innate immune cells such as neutrophils to the site of inflammation [1,2]. Besides its potent chemotactic activity, IL-8 activates cells by induction of respiratory burst, exocytosis and degranulation of storage proteins, which are involved in wound healing and inflammation [1,3–5]. IL-8 also promotes proliferation, growth, and viability of vascular endothelial cells, which are involved in angiogenesis [6,7]. IL-8 was initially purified from human blood monocytes stimulated with lipopolysaccharide (LPS) [8,9]. However, this chemokine is recognized to be produced and secreted by various cell types including non-immune cells, like fibroblasts and endothelial cells [10–12].

IL-8 expression is almost undetectable in unstimulated cells but is rapidly stimulated by a variety wide range of stimuli including pro-inflammatory cytokines [12]. Interleukin-1β (IL-1β) is a pro-inflammatory cytokine, which mediates expression and secretion of cytokines and chemokines in inflamed tissues and contributes to acute or chronic inflammation [13,14]. IL-1β has been demonstrated to mediate the expression and secretion of IL-8 [15–18].

Mitogen-activated protein kinases (MAPKs) are important regulatory enzymes and are activated by a variety of stimuli, including pro-inflammatory cytokines such as IL-1β and tumor necrosis factor α (TNF-α) [19]. There are three major MAPKs in mammalians: extracellular signal-regulated kinases 1/2 (ERK1/2), c-Jun N-terminal kinases (JNK), and p38 MAPK [19,20]. The activation of MAPKs has been demonstrated to contribute to IL-1β-induced IL-8 expression in various human cells [21–26].

Tumor progression locus 2 (Tpl2), also known as Cot, is a member of the MAP3K family of serine/threonine kinases involved in innate and adaptive immunity and inflammation [27–29]. Tpl2 contributes to the optimal functions in cells in response to various stimuli including cytokines [27–30]. Although the roles for Tpl2 in immune cells (i.e. macrophages and neutrophils) were reported, the contribution of Tpl2 in sentinel immune system (i.e. fibroblasts) has been obscure [31–34].

Dermal wound healing is an intricate process, but an essentially physiological process required to restore the integrity of skin after trauma such as acute injury and surgery. The process involves a series of sequential and overlapping phases such as hemostasis, inflammation, proliferation, and remodeling [35–38]. These phases are tightly regulated by the timing of expression and secretion of the abundance of cytokines and growth factors in various cells, such as the cells consisted of dermis and epidermis, and resident and circulating immune cells [30,39]. Dermal fibroblasts act as a sentinel immune system during inflammatory states and dermal wound healing [40]. Dermal fibroblasts produce and secrete a variety of cytokines, chemokines and growth factors during the inflammatory phase, which synchronize the migration of immune cells to the wound bed and regulate their retention and survival in damaged tissue [41,42].

Although several mouse models have been established to investigate the pathophysiology and pharmacological treatments for atopic dermatitis, it has been difficult to accurately simulate the feature of human atopic dermatitis using single mouse model [43]. On the other hand, canine atopic dermatitis is a naturally occurring disease which is similar to human. Like human’s, canine atopic dermatitis is characterized by the upregulation of inflammatory genes (i.e., COX-2, IL-6 and IL-8) and Th2 cytokines (i.e., IL-4, IL-5, IL-13, IL-31 and IL-33) [44,45]. Therefore, dogs could be a pathological and pharmacological model for atopic dermatitis in human. However, the mechanism of inflammation in canine dermal fibroblasts has been unclear.

In this study, we demonstrate that IL-1β stimulates IL-8 expression via ERK1/2 activation and Tpl2 contributes to IL-1β-mediated ERK1/2 activation in canine dermal fibroblasts.

## Materials and methods

### Materials

α-Modified Eagle minimum essential medium (α-MEM), sodium fluoride, and 4-(2-hydroxyethyl)-1-piperazineethanesulfonic acid (HEPES), and phenylmethanesulfonyl fluoride (PMSF) were purchased from Wako Pure Chemical Industries, Ltd. (Osaka, Japan). Recombinant canine IL-1β was purchased from Kingfisher Biotech, Inc. (Saint Paul, MN). TRIzol and Lipofectamine 2000 were obtained from Life Technologies Co. (Carlsbad, CA). Thermal Cycler Dice Real Time System II, TP900 Dice Real Time v4.02B, SYBR Premix Ex Taq II, PrimeScript RT Master Mix, and CELLBANKER 1 plus medium were obtained from TaKaRa Bio Inc. (Shiga, Japan). FR180204, SP600125, SB239063, SKF86002, anti-β-actin mouse monoclonal antibody (AC74, Cat# A5441, RRID:AB_476744), siRNA for Tpl2, ERK1 and ERK2, and scramble siRNA were obtained from Sigma-Aldrich Inc. (St Louis, MO). Rabbit monoclonal antibodies against rat total-ERK1/2 (t-ERK1/2, 137F5, Cat# 4695, RRID:AB_390779) and human phospho-ERK1/2 (p-ERK1/2, D13.14.4E, Cat# 4370, RRID:AB_2315112) were purchased from Cell Signaling Technology Japan, K.K. (Tokyo, Japan). Horseradish peroxidase (HRP)-conjugated anti-mouse and anti-rabbit IgG antibodies, ECL Western Blotting Analysis System, and ImageQuant LAS 4000 mini were purchased from GE Healthcare (Piscataway, NJ). Rabbit polyclonal antibodies against human Tpl2 (t-Tpl2, Cat# ab49152, RRID:AB_2297369) was purchased from Abcam (Cambridge, UK). Polyvinylidene difluoride (PVDF) membranes and Mini-PROTEAN TGX gel were obtained from Bio-Rad (Hercules, CA). Complete mini EDTA-free protease inhibitor mixture and Block Ace were purchased from Roche (Mannheim, Germany). An enzyme-linked immunosorbent assay (ELISA) kit for canine IL-8, a freezing vessel (BICELL), and StatMate IV were purchased from R&D Systems, Inc. (Minneapolis, MN), Nihon Freezer Co., Ltd. (Tokyo, Japan), and ATMS (Tokyo, Japan), respectively.

### Cell culture

Three healthy beagles (male, 3 years old) were purchased from Japan SLC Inc., and bred and maintained in cages (height: 137 cm; width: 80 cm; length: 86 cm). The experimental food TC-2 (250 g/head; Oriental Yeast Co. Ltd.) was provided to all study animals once a day. The dogs were exercised using some toys inside (once a day) and outside (once a month) of the animal breeding facility. The physical conditions of the facility were monitored once a day. To avoid infection, the dogs were housed distantly from each other. All efforts were made to improve animal welfare and minimize discomfort. The dogs used in this research were housed for use in further research. This study was approved by Nihon University Animal Care and Use Committee (AP13B051). After local anesthesia with 1% lidocaine and 10 g/mL adrenaline, dog dorsal skin samples were collected. To relieve pain after the procedure, butorphanol tartrate (0.2 mg/kg) was administered intravenously. Canine dermal fibroblasts were isolated by explant culture using a method previously described [46–50] with slightly modifications. Cells were characterized by detecting the mRNA expression of chemotropic factors such as: Netrin-1, Netrin-3, Ephrin-A3, Ephrin-A4, and Semaphorin-4D as reported previously [47]. The mRNA expression of chemotropic factors in dermal fibroblasts was lower compared to mesenchymal stem cells, confirming that the cells are dermal fibroblasts. Briefly, canine dermis collected from the dorsal skin was cut into 3-mm2 sections and placed into 90-mm Petri dish. The attached explants were retained in a static-culture in an incubator at 5% CO2 and 37°C using α-MEM supplemented with 10% fetal bovine serum (FBS), and the medium was changed once a week. After then, canine dermal fibroblasts were obtained as outgrowth cells. When canine dermal fibroblasts reached 90–95% confluence, they were harvested using 0.25% trypsin-EDTA. The fibroblasts collected were suspended using CELLBANKER 1 plus medium (Takara Bio Inc., Shiga, Japan) at a density of 2 × 10^6^ cells/500 μL, divided into 500 μL each, and placed into a sterilized serum tube. The tubes were then placed into the freezing vessel BICELL and cryopreserved at −80°C. Before use in experiments, serum tubes were taken out from the BICELL vessel and immersed into a water bath at 37°C. The thawed-out cell suspension was transferred into a centrifuge tube contained α-MEM containing 10% FBS. After centrifugation at 300 × g for 3 min and subsequent removal of the supernatant, the pellet was suspended in α-MEM containing 10% FBS and transferred into a 75-cm^2^ culture flask. Static cultures were then performed under the same conditions as before the cryopreservation. Fibroblasts were harvested using 0.25% trypsin-EDTA once they reached approximately 90% confluence and seeded at a density of 1 × 10^6^ cells per 75-cm^2^ culture flask. The fourth-passage fibroblasts were used for all following experiments. An experimental result using cells derived from one dog was taken as one case.

### Real-time polymerase chain reaction (RT-PCR)

Real-time RT-PCR was performed as previously reported [47–60]. Total RNA extraction from cultured canine dermal fibroblasts was performed using TRIzol following the manufacturer’s instructions. Synthesis of cDNA was carried out with 500 ng of total RNA using PrimeScript RT Master Mix. Real-time RT-PCR was performed with 2 μL of first-strand cDNA in 25 μL (total reaction volume), SYBR Premix Ex Taq II, and primers targeting canine IL-8 or the TATA box binding protein (TBP), as the housekeeping gene (Table 1). Real-time RT-PCRs of “no-template” controls or “no-reverse transcription” controls were performed with 2 μL of RNase- and DNA-free water or 2 μL of each RNA sample, respectively. PCR was performed using Thermal Cycler Dice Real Time System II. The protocol was as follows: 1 cycle of denaturation at 95°C for 30 sec, 40 cycles of denaturation at 95°C for 5 sec, and annealing/extension at 60°C for 30 sec. Results were analyzed by the second derivative maximum method and the comparative cycle threshold (ΔΔCt) method, using real-time RT-PCR analysis software. The amplification of TBP from the same amount of cDNA was applied as an endogenous control, while cDNA amplification from canine dermal fibroblasts at time 0 was used as the calibration standard.

**Table 1:** Primer sequences for RT-qPCR.

Gene Name	Gene bank ID	Primer sequences
*IL-8*	NM_001003200.1	F: 5ʹ- CACCTCAAGAACATCCAGAGCT -3ʹ
		R: 5ʹ- CAAGCAGAACTGAACTACCATCG -3ʹ
*TBP*	XM_863452	F: 5’-ACTGTTGGTGGGTCAGCACAAG-3’
		R: 5’-ATGGTGTGTACGGGAGCCAAG-3’

### Western blotting

Western blotting was performed as previously described [47–59]. Cells were lysed with 20 mM HEPES buffer (pH 7.4) containing 1 mM PMSF, 10 mM sodium fluoride and a complete mini EDTA-free protease inhibitor cocktail. Protein concentrations were determined by the Bradford method [61] and adjusted. After boiled at 98°C for 5 min in SDS buffer, extracted protein samples were loaded into separate lanes of 12% Mini-PROTEAN TGX gel and electrophoretically separated. Separated proteins were transferred to PVDF membranes, treated with Block Ace for 50 min at room temperature, and incubated with primary antibodies [p-ERK1/2 (1:1,000), t-ERK1/2 (1:1,000), Tlp2 (1:1,000), and β-actin (1:10,000)] for 120 min at room temperature. After washing, membranes were incubated with HRP-conjugated anti-rabbit or anti-mouse IgG (1:10,000) for 90 min at room temperature. Immunoreactivity was detected using ECL Western Blotting Analysis System, and chemiluminescent signals of membranes were measured using ImageQuant LAS 4000 mini.

### IL-8 ELISA

Canine dermal fibroblasts were seeded at a density of 3.0 × 10^5^ cells/well in 6-well culture plates. After starvation for 24 h, fibroblasts were treated with IL-1β for 0–24 h, and the culture medium was collected. IL-8 concentration in the culture medium was assayed using an ELISA kit according to the manufacturer’s instructions.

### Transfection of siRNA

siRNA transfection was performed as previously described [47–55,57–59]. Canine dermal fibroblasts were seeded at a density of 1 × 10^5^ cells/35 mm dish or 5 × 10^5^ cells/90 mm dish, and transfected using Opti-MEM containing 10 μL/mL Lipofectamine 2000 and 100 nM siRNA of ERK1, ERK2 or Tpl2, or scramble siRNA for 6 h (Table 2). After the transfection, the medium was changed to α-MEM containing 10% FBS, and the cultures were maintained in an incubator with 5% CO_2_ at 37°C for 5 days.

**Table 2:** Sequences for siRNA transfection.

Gene Name	Gene bank ID	siRNA sequences
*ERK1*	NM_001252035.1	CCAATGTGCTCCACCGGGA
*ERK2*	NM_001110800.1	CCCAAATGCTGACTCGAAA
*Tpl2* #1	XM_005617057.3	GAAAGTGATTCATCATGAT
*Tpl2* #2	XM_005617057.3	GAGAACATCGCTGAGTTAT

### Statistical analysis

Statistical analyses were performed using StatMate IV, and data from all experiments are presented as the mean ± standard error. Data from the time-course study and other experiments were analyzed using two-way analysis of variance (ANOVA) and one-way ANOVA, respectively. Tukey’s test was used as post-hoc analysis. *P*-values inferior to 0.05 were considered statistically significant.

## Results

### IL-1β-induced secretion of IL-8 via the induction of IL-8 expression in dermal fibroblasts

**Fig 1:**
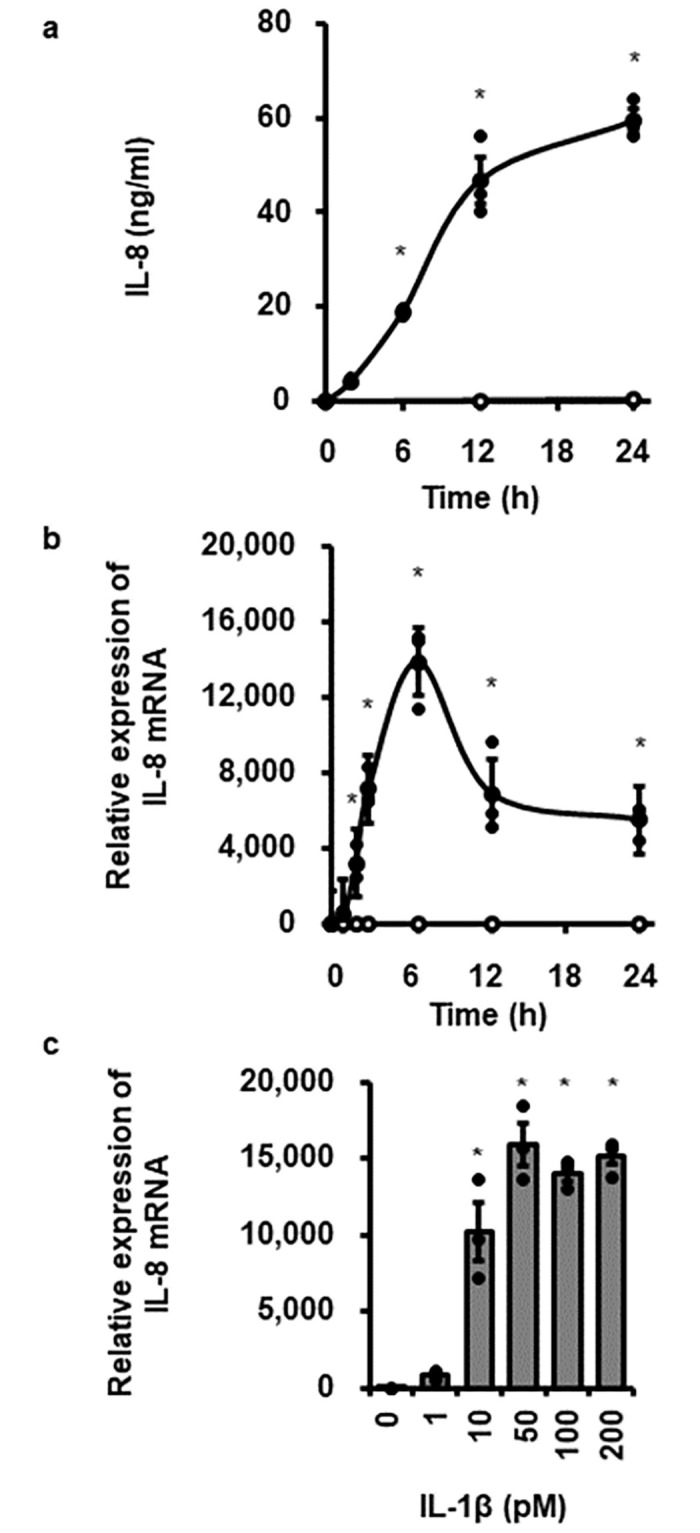
IL-1β-induced IL-8 release and IL-8 mRNA expression in canine dermal fibroblasts. Time-dependent increase in IL-8 protein release (a) and IL-8 mRNA expression levels (b) in cells treated with (closed circle) or without (open circle) canine recombinant IL-1β (100 pM). (c) Dose-dependent increase in IL-8 mRNA expression levels in cells treated with the indicated concentrations of IL-1β for 6 h. TBP was used as an internal standard and the expression levels of IL-8 mRNA in IL-1β-stimulated cells were compared with the expression at 0 h. Results have been represented as mean ± standard error (SE) from biological triplicates. *P<0.05.

When canine dermal fibroblasts were incubated with 100 pM IL-1β for 0–24 h, the concentration of IL-8 in the incubation medium was increase in a time-dependent manner (Fig 1a). Then, we examined the effect of IL-1β on IL-8 mRNA expression in canine dermal fibroblasts. IL-8 mRNA expression was time-dependently increased, reached at 6 h, and then decreased (Fig 1b). In cells treated with various concentrations of IL-1β (0–200 pM) for 6 h, IL-1β enhanced IL-8 mRNA expression in a dose-dependent manner and above 50 pM reached a plateau level (Fig 1c). These observations suggest that IL-1β provokes IL-8 expression and secretion in canine dermal fibroblasts.

### Involvement of MAPKs in IL-1β-induced IL-8 expression

**Fig 2:**
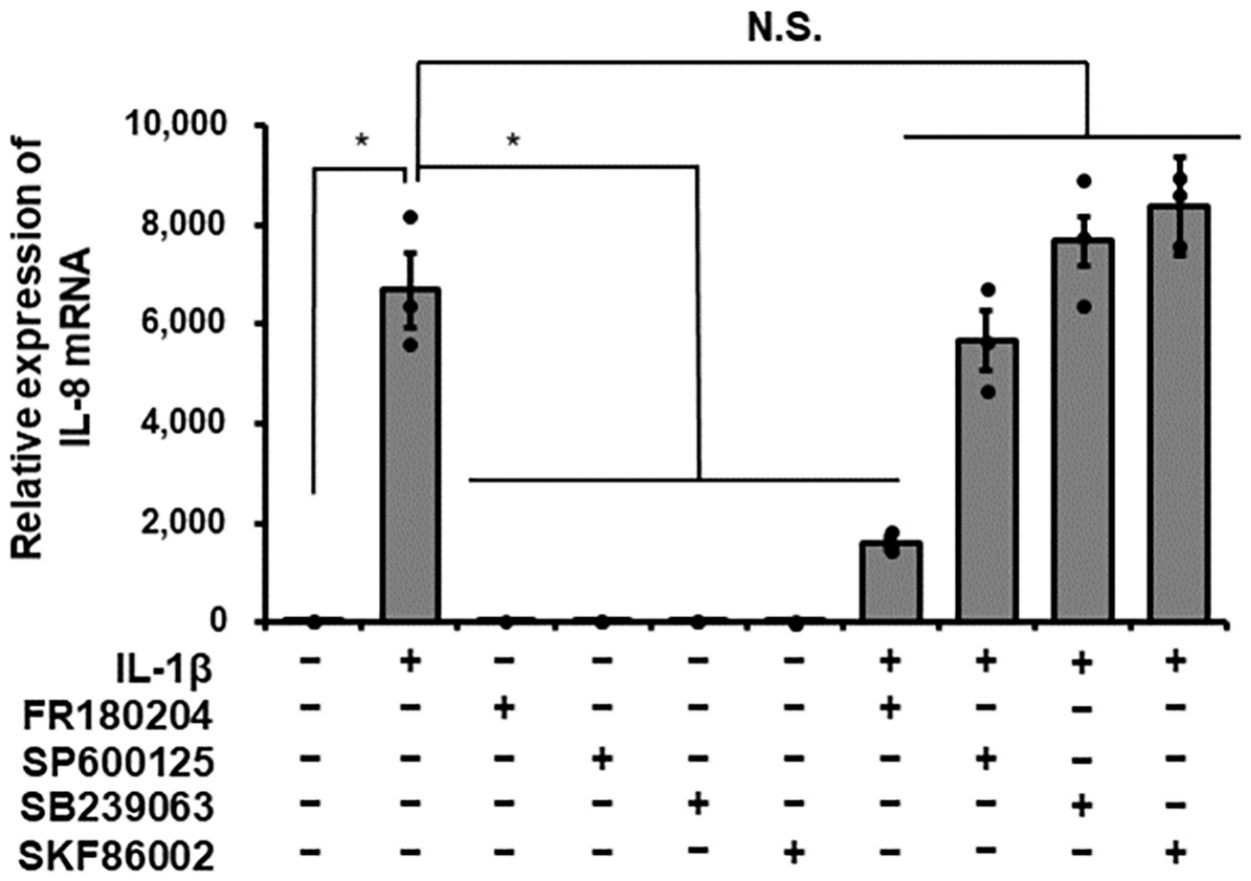
Effect of MAPK inhibitors on IL-1β-induced IL-8 mRNA expression. Canine dermal fibroblasts were pretreated with or without the ERK1/2 inhibitor FR180204 (50 μM), JNK inhibitor SP600125 (10 μM), and p38 inhibitors SB239063 (10 μM) and SKF86002 (10 μM) for 1 h and subsequently stimulated with or without IL-1β (100 pM) for 6 h. After stimulation, IL-8 mRNA expression levels were determined. TBP was used as an internal standard and the expression levels of IL-8 mRNA in IL-1β-stimulated cells were compared with the expression at 0 h. Results have been represented as mean ± SE from biological triplicates. *P<0.05.

**Fig 3:**
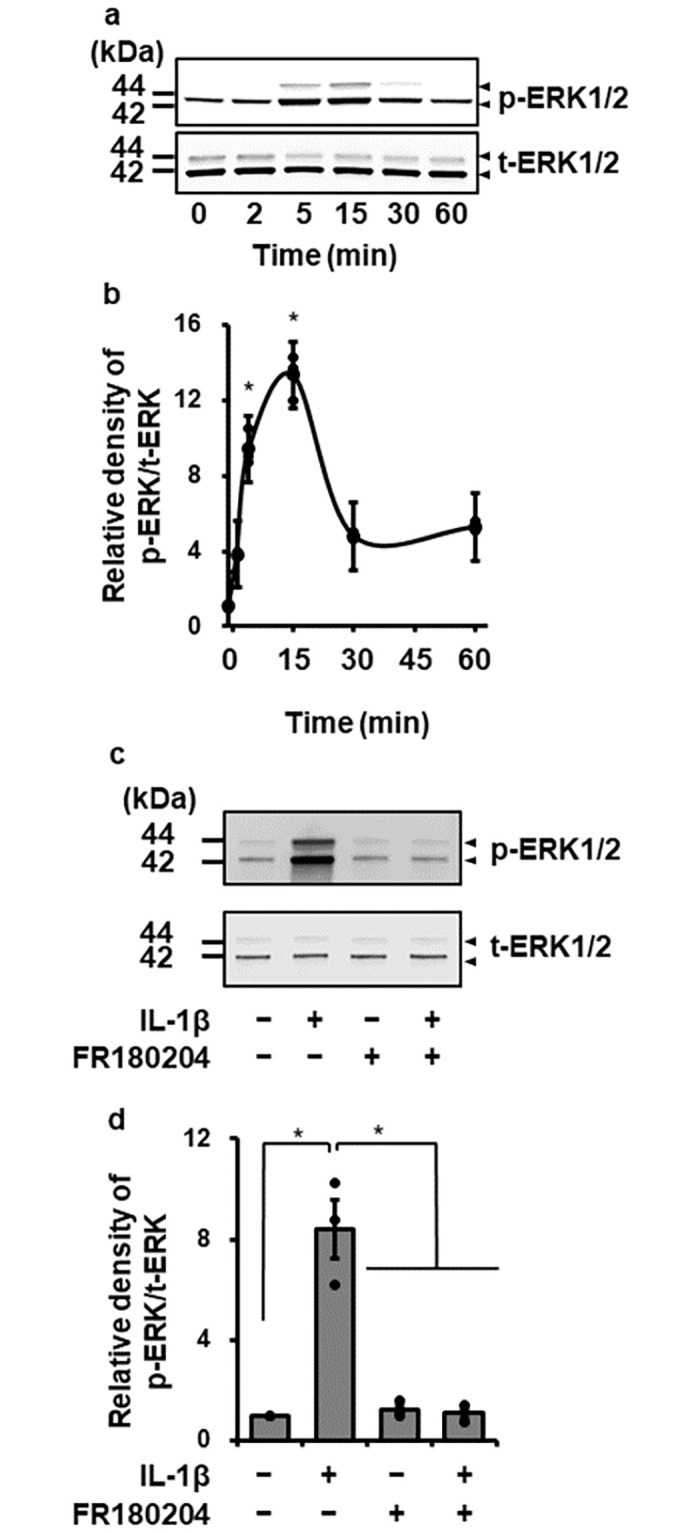
IL-1β-induced activation of ERK1/2. (a, b) Time-dependent changes of ERK1/2 phosphorylation. Western blotting for the levels of phosphorylated ERK1/2 (p-ERK1/2) and total ERK1/2 (t-ERK1/2) in dermal fibroblasts treated with IL-1β (100 pM) (a) and relative levels of [p-ERK1/2]/[t-ERK1/2] in IL-1β-stimulated cells compared to the levels at 0 h (b). (c, d) Effect of an ERK1/2 inhibitor on IL-1β-mediated ERK1/2 phosphorylation. Fibroblasts were pretreated with or without the ERK1/2 inhibitor FR180204 (50 μM) for 1 h and stimulated with IL-1β for 15 min. Representative Western blotting result of inhibitory effect of the ERK1/2 inhibitor on IL-1β-mediated ERK1/2 phosphorylation (c) and relative levels of [p-ERK1/2]/[t-ERK1/2] as compared to those without the inhibitor and IL-1β (d). Results have been represented as mean ± SE from biological triplicates. *P<0.05.

To investigate the contribution of MAPK signaling pathways in IL-1β-induced IL-8 production, we examined the effect of MAPK inhibitors on IL-1β-induced IL-8 mRNA expression. The cells were pretreated with 25 μM FR180204 (for ERK1/2), 10 μM SP600125 (for JNK), 20 μM SB239063 or 20 μM SKF86002 (both for p38 MAPK) for 1 h and then stimulated with 100 pM IL-1β for 6 h. As Fig 2a shows, the ERK1/2 inhibitor FR180204 clearly inhibited IL-1β-induced IL-8 mRNA expression, but the JNK inhibitor and the p38 MAPK inhibitors did not. Next, we examined the effect of IL-1β on ERK1/2 phosphorylation in canine dermal fibroblasts. When cells were stimulated with 100 pM IL-1β for 0–60 min, ERK1/2 phosphorylation was observed at 5–15 min after stimulation, indicating the activation of ERK1/2 by IL-1β (Fig 3a and 3b). In cells pretreated with the ERK inhibitor FR180204 for 1 h, IL-1β-mediated ERK1/2 phosphorylation was clearly attenuated (Fig 3c and 3d). These observations suggest that IL-1β induces IL-8 expression via ERK1/2 activation.

**Fig 4:**
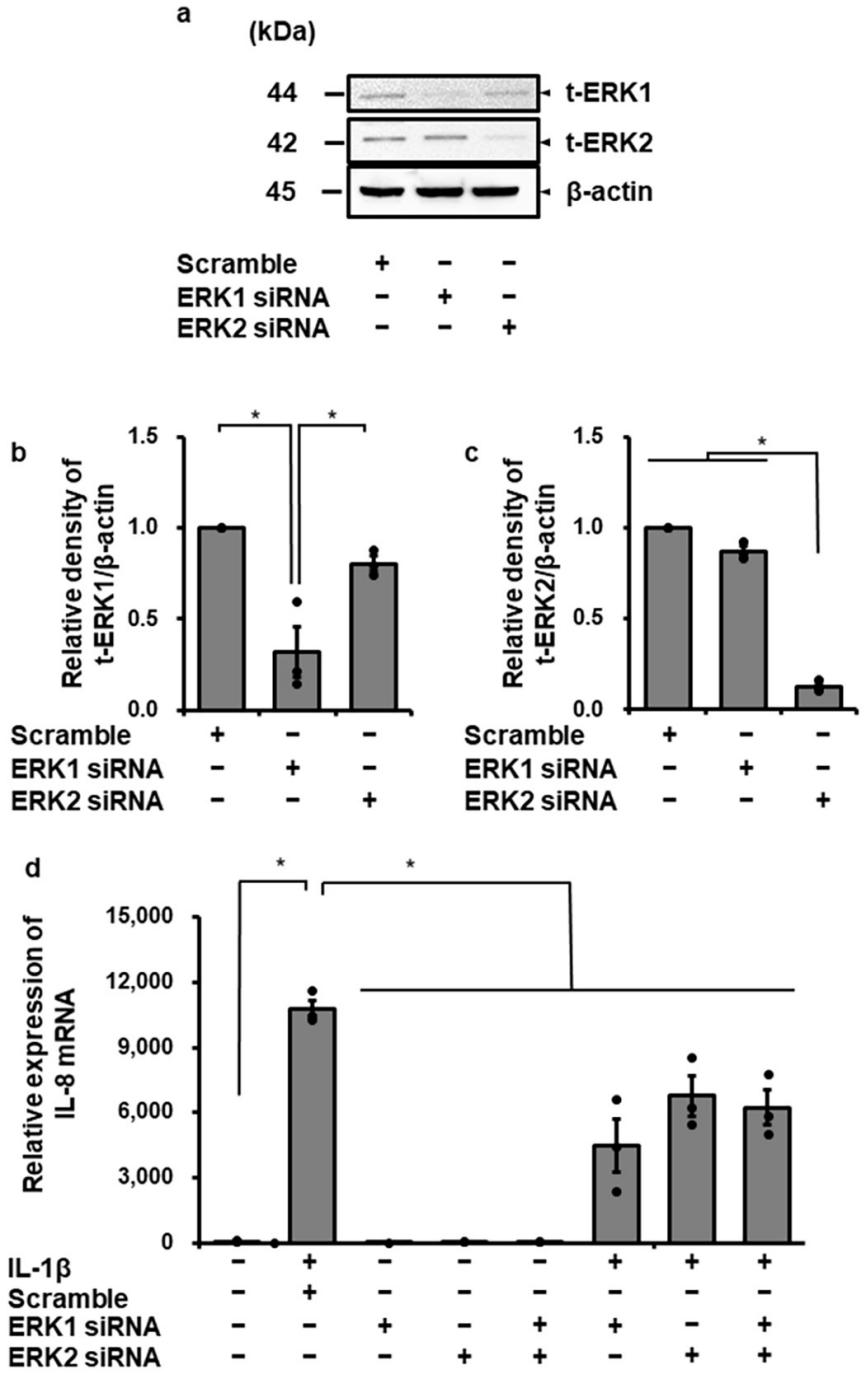
Attenuation of IL-1β-induced IL-8 mRNA expression in canine dermal fibroblasts transfected with ERK1and ERK2 siRNAs. (a-c) In canine dermal fibroblasts transfected with ERK1, ERK2, and scrambled siRNAs, expression of t-ERK1, t-ERK2, and β-actin was detected by western blotting. The expression of ERK1 or ERK2 was reduced in cells transfected with ERK1 or ERK2 siRNA, respectively, but not in cells transfected with scrambled siRNA. β-actin was used as an internal standard. Representative results (a) and relative density of t-ERK1 or ERK2 protein expression in siRNA-transfected cells compared with those in scrambled siRNA-transfected cells (b, c) are depicted. (d) Dermal fibroblasts transfected with ERK1, ERK2 and scrambled siRNAs were incubated with or without IL-1β (100 pM) for 6 h. After the incubation, IL-8 mRNA expression was determined. TBP was used as an internal standard. ERK1 and ERK2 siRNA transfection resulted in reduction of IL-1β-induced IL-8 mRNA expression, while scrambled siRNA-transfection did not. In ERK1 and 2 double knockdown cells, IL-1β-induced IL-8 mRNA expression was also attenuated. Results have been represented as mean ± SE from biological triplicates. *P<0.05.

To confirm the contribution of ERK1/2 to IL-1β-induced IL-8 expression, we examined the effect of IL-1β on IL-8 mRNA expression in ERK1/2 knockdown cells using siRNA transfection. In cells transfected with ERK1 or ERK2 siRNA, ERK1 or ERK2 protein expression was clearly reduced, respectively, compared with that in control cells transfected with scramble RNA (Fig 4a–4c). In cells transfected with ERK1, ERK2 or both ERK1 and ERK2 siRNAs, IL-1β-induced IL-8 mRNA expression clearly decreased compared with control, although no additive effect of ERK1 and ERK2 siRNA was shown (Fig 4d). Taken together, it is most likely that IL-1β induces IL-8 expression via ERK1/2 activation in canine dermal fibroblasts.

### Tpl2 contributes to IL-1β-induced IL-8 expression via ERK1/2 activation

**Fig 5:**
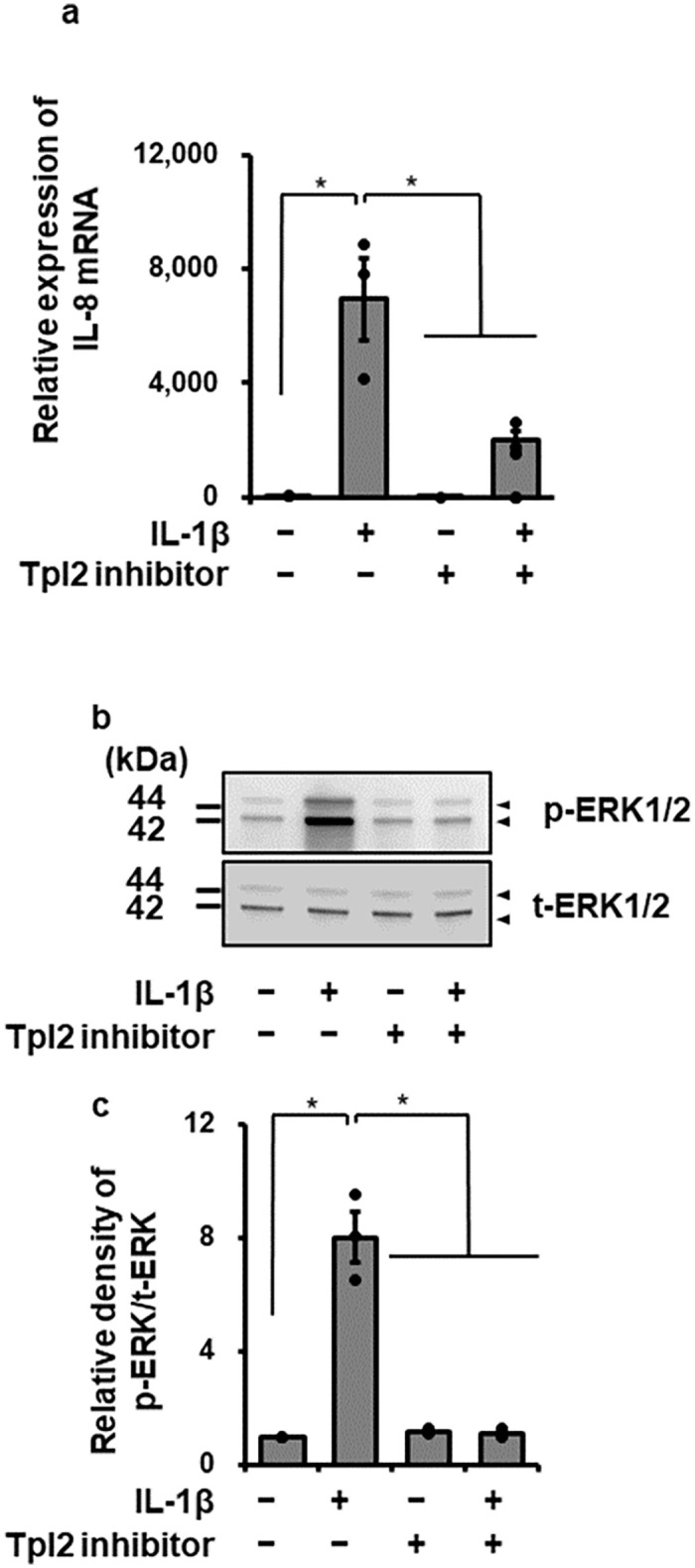
Inhibitory effect of a Tpl2 inhibitor on IL-1β-induced IL-8 mRNA expression and ERK1/2 phosphorylation in canine dermal fibroblasts. Canine dermal fibroblasts were pretreated with or without Tpl2 kinase inhibitor II (10 μM) for 1 h and subsequently stimulated with or without IL-1β (100 pM). (a) After stimulation for 6 h, IL-8 mRNA expression levels were determined. TBP was used as an internal standard and the expression levels of IL-8 mRNA in IL-1β-stimulated cells were compared with the expression at 0 h. (b, c) After stimulation for 15min, phosphorylated ERK1/2 (p-ERK1/2) and total ERK1/2 (t-ERK1/2) were detected by western blotting. Representative western blotting result of inhibitory effect of the Tpl2 inhibitor on IL-1β-mediated ERK1/2 phosphorylation (b) and relative levels of [p-ERK1/2]/[t-ERK1/2] as compared to those without the inhibitor and IL-1β (c). Results have been represented as mean ± SE from biological triplicates. *P<0.05.

ERK1/2 MAPK has been reported to be regulated with Tpl2 in IL-1β-stimulated cells [28,30]. Then, effect of a Tpl2 inhibitor on IL-1β-induced IL-8 mRNA expression was examined. As Fig 5a summarizes, in cells pretreated with Tpl2 kinase inhibitor II (10 μM), a Tpl2 inhibitor, for 1 h, IL-1β-induced IL-8 mRNA expression was clearly reduced. The Tpl2 inhibitor also attenuated the effect of IL-1β on ERK1/2 phosphorylation, as shown in Fig 5b and 5c. This observation suggests that Tpl2/ERK1/2 signaling pathway contributes to IL-1β-induced IL-8 mRNA expression.

**Fig 6:**
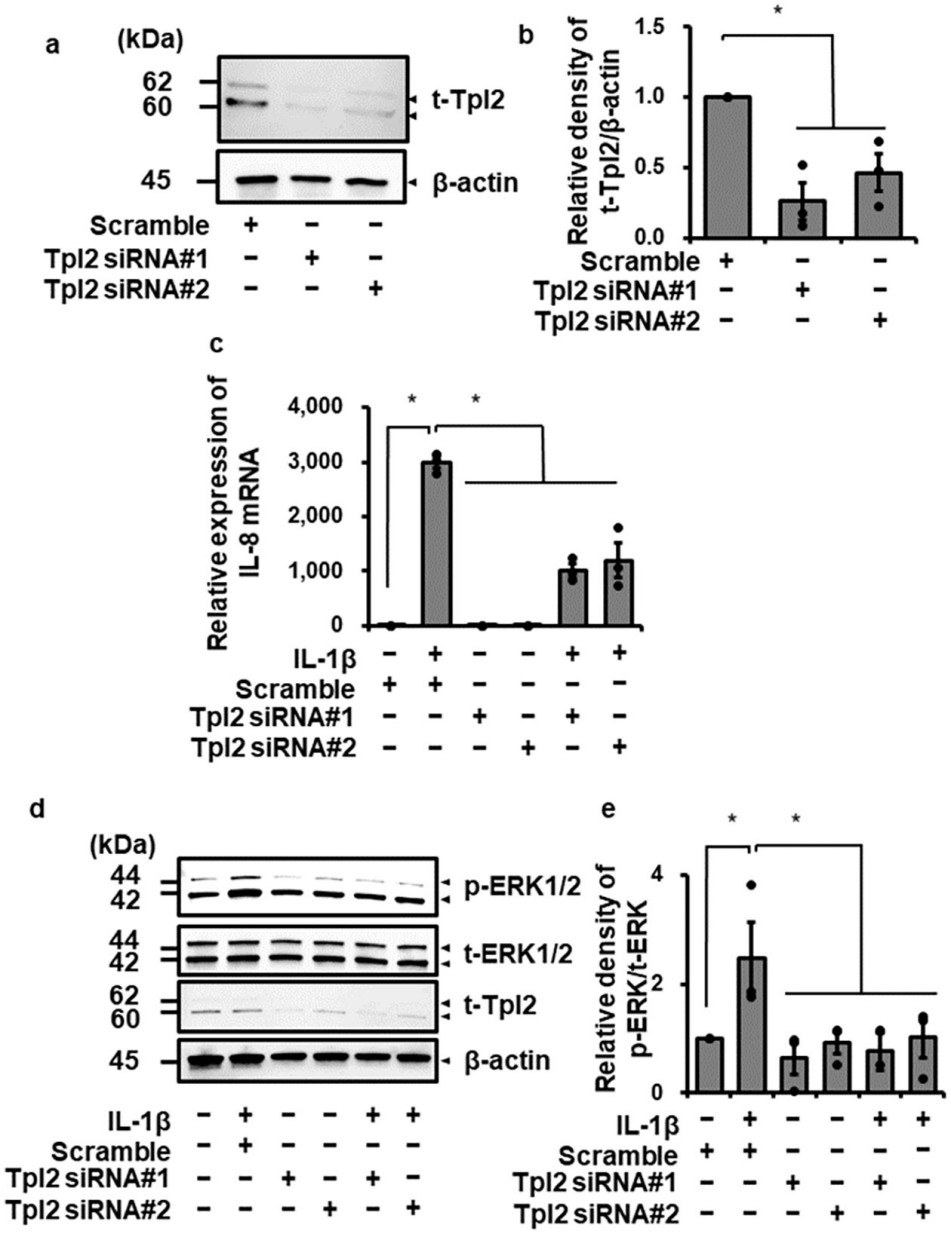
Attenuation of IL-1β-induced IL-8 mRNA expression in canine dermal fibroblasts transfected with Tpl2 siRNAs. (a, b) In canine dermal fibroblasts transfected with two kinds of Tpl2 siRNA or scrambled siRNA, expression of total Tpl2 (t-Tpl2) and β-actin was detected by western blotting. The expression of t-Tpl2 was reduced in cells transfected with both Tpl2 siRNAs, but not in cells transfected with scrambled siRNA. β-actin was used as an internal standard. Representative results (a) and relative density of t-Tpl2 expression, shown as [t-Tpl2//β-actin], in siRNA-transfected cells compared with those in scrambled siRNA-transfected cells (b) are depicted. (c) Canine dermal fibroblasts transfected with Tpl2 and scrambled siRNAs were incubated with or without IL-1β (100 pM) for 6 h. After the incubation, IL-8 mRNA expression was determined. TBP was used as an internal standard. Transfection with Tpl2 siRNAs resulted in attenuation of IL-1β-induced IL-8 mRNA expression, while scrambled siRNA-transfection did not. (d, e) Fibroblasts transfected with Tpl2 and scrambled siRNAs were stimulated with or without IL-1β for 15 min. After the stimulation, phosphorylated ERK1/2 (p-ERK1/2), total ERK1/2 (t-ERK1/2), t-Tpl2 and the internal standard β-actin were detected by western blotting. The expression of p-ERK1/2 was reduced in Tpl2-knockdown cells, but not in scrambled siRNA-transfected cells. No change of t-ERK1/2 expression was observed in all cells. Representative results (d) and relative density of [p-ERK1/2/t-ERK1/2] in Tpl2 siRNA-transfected cells compared with those in scrambled siRNA-transfected cells (e) are depicted. Results have been represented as mean ± SE from biological triplicates. *P<0.05.

In canine dermal fibroblasts, the phosphorylation of Tpl2 Ser400 and Thr290 could not be detected (S1 Fig). On the other hand, IL-1β induced the phosphorylation of MEK (S2a Fig), but the MEK inhibitor U0126 failed to attenuate the IL-1β-induced IL-8 expression (S2b Fig). Since the understanding of how phosphorylation regulates Tpl2 activation has been unclear [62], we investigated the contribution of Tpl2 to IL-1β-induced IL-8 expression and ERK1/2 activation using siRNA specific for Tpl2. In cells transfected with two kinds of Tpl2 siRNA, total Tpl2 protein expression was significantly reduced (Fig 6a and 6b). In the Tpl2 knockdown cells, IL-1β-mediated IL-8 mRNA expression and ERK1/2 phosphorylation were clearly attenuated, as Fig 6c, 6d and 6e, respectively. Taken together, it is most likely that Tpl2 acts as a regulator of ERK signaling, and which contributes to IL-1β-mediated IL-8 expression in canine dermal fibroblasts.

## Discussion

We demonstrated here that IL-1β stimulated mRNA expression and protein secretion of IL-8 in canine dermal fibroblasts. The increase in serum concentrations or protein expression of IL-8 was observed in dogs with spontaneously occurred inflammatory diseases and cancers [63–69] and inflammatory model treated with LPS [70,71]. The expression of IL-8 mRNA increased in canine endometrial stromal cells [72] and synovial fibroblasts stimulated with LPS and the pro-inflammatory cytokine TNF-α [54], respectively. In human, elevated IL-8 expression has been attributed to a number of diseases such as chronic obstructive pulmonary disease, hypertension, carcinogenesis, idiopathic pulmonary fibrosis and chronic periodontitis [68]. These observations strongly suggest that IL-1β-induced IL-8 is involved in systematic and local inflammation. Therefore, it is most likely that IL-1β-induced IL-8 contributes to dermal inflammation.

The activation of MAPKs has been demonstrated to contribute to IL-1β-induced IL-8 expression. However, the activation is cell context specific. In human bronchial epithelial cells [21] and myometrial cells [22] p38 MAPK and ERK are involved in IL-1β-induced IL-8 expression, respectively. In Hela cells [23] and human Müller cells [24], both ERK and p38 MAPK contribute to IL-1β-mediated IL-8 expression. In HepG2 cells [25] and human ovarian granulosa cells [26], IL-1β stimulates IL-8 expression via the activation of both JNK and p38 MAPK. In canine dermal fibroblasts, IL-1β stimulated ERK1/2 phosphorylation. The effect of IL-1β on ERK1/2 activation and IL-8 expression was inhibited by a specific pharmacological inhibitor for ERK1/2. IL-1β-induced ERK1/2 activation and IL-8 expression were also reduced in cells transfected with siRNA of ERK1 and ERK2. These observations strongly suggest that ERK1/2 signaling pathway contributes to IL-1β induces IL-8 expression in canine dermal fibroblasts.

The isoforms of ERK, ERK1 and ERK2, seem to be co-expressed ubiquitously and generally coactivated in cells stimulated with multiple extracellular stimuli [69,73]. We previously demonstrated functional difference between ERK1 and ERK2 in canine dermal fibroblasts [74] as well as in canine and feline synovial fibroblasts [54,75] by ERK-knockdown experiments by treatment with ERK isoform-specific siRNA. In this study, we examined effect of IL-1β on IL-8 mRNA expression in ERK1- and/or ERK2-knockdown cells. IL-1β-induced IL-8 mRNA expression was reduced in each ERK1- or ERK2-knockdown cells or the ERK1/ERK2 co-knockdown cells. However, the reduction of IL-1β on IL-8 mRNA expression in the co-knockdown cells showed no significant difference from that in the single ERK1 or ERK2 knockdown cells. These observations imply functional redundancy of ERK1 and ERK2 pathways in IL-1β-induced IL-8 expression in canine dermal fibroblasts, as well as shown in various tissues [76].

The roles for Tpl2 in central immune system during autoimmune and infectious diseases has been investigated. In Tpl2-deficient mice, the protection from numerous inflammatory and autoimmune diseases has been observed. The contribution of Tpl2 in IL-1β response has been reported in mouse macrophages [39,75] and human monocytes [75]. However, in Rag1^-/-^Tpl2^-/-^ mice, which lacks mature B and T lymphocytes, the relative concentrations of circulating immune cells were increased during the infection with Staphylococcus xylosus, compared with Rag1^-/-^ mice [31]. In this study, we demonstrated the involvement of Tpl2 in IL-1β-induced IL-8 expression in canine dermal fibroblasts, since IL-1β failed to induce IL-8 mRNA in cells pretreated with a Tpl2 inhibitor and transfected with Tpl2 siRNAs. These observations support the notion that Tpl2 plays an important role in sentinel immune system (i.e. fibroblasts). In our study, IL-1β-mediated ERK1/2 phosphorylation was attenuated in the Tpl2 inhibitor-treated cells and Tpl2 knockdown cells. In human synovial fibroblasts with rheumatoid arthritis, Tpl2 also induced ERK activation in the presence of IL-1β [76], whereas IL-1β stimulated the activation of ERK but also p38 MAPK signaling and JNK signaling in HeLa epithelial cell line or rat INS-1E β-cells, respectively [23,76]. These observations suggest that Tpl2 contributes to activation of ERK1/2 signaling pathway of fibroblasts in sentinel immune system. Studies of precise mechanisms with Tpl2 activation by IL-1β stimulation and ERK1/2 signaling activation by Tpl2 are underway in our laboratory.

## Conclusion

Since atopic dermatitis is less responsive to the current therapeutic approach, our observations highlight the role of Tpl2/ERK1/2 signaling pathway as a promising therapeutic target in canine skin inflammation, such as atopic dermatitis.

## Supporting information

S1 Fig The effect of IL-1β on the phosphorylation of Tpl2. When cells were stimulated with 100 pM IL-1β for 0–60 min, the phosphorylation of Tpl2 Ser400 and Thr290 could not be detected.
(PDF)

S2 Fig The contribution of MEK activation to IL-1β-induced IL-8 mRNA expression. (a) When cells were stimulated with 100 pM IL-1β for 0–60 min, the phosphorylation of MEK was observed. (b) Canine dermal fibroblasts were pretreated with or without the MEK inhibitor U0126 (10 μM) for 1 h and subsequently stimulated with or without IL-1β (100 pM) for 6 h. After stimulation, IL-8 mRNA expression levels were determined. TBP was used as an internal standard. Results have been represented as mean ± SE from biological triplicates. *P < 0.05.
(PDF)

S1 Raw images (PDF)
