# Supplementary material for: Tpl2 contributes to IL-1β-induced IL-8 expression via ERK1/2 activation in canine dermal fibroblasts
Source: PLoS One. 2021 Nov 4;16(11):e0259489. doi: 10.1371/journal.pone.0259489 (PMC8568182; doi:10.1371/journal.pone.0259489)
Supplement: S1 Fig — When cells were stimulated with 100 pM IL-1β for 0–60 min, the phosphorylation of Tpl2 Ser400 and Thr290 could not be detected. (PDF) [file pone.0259489.s001.pdf]

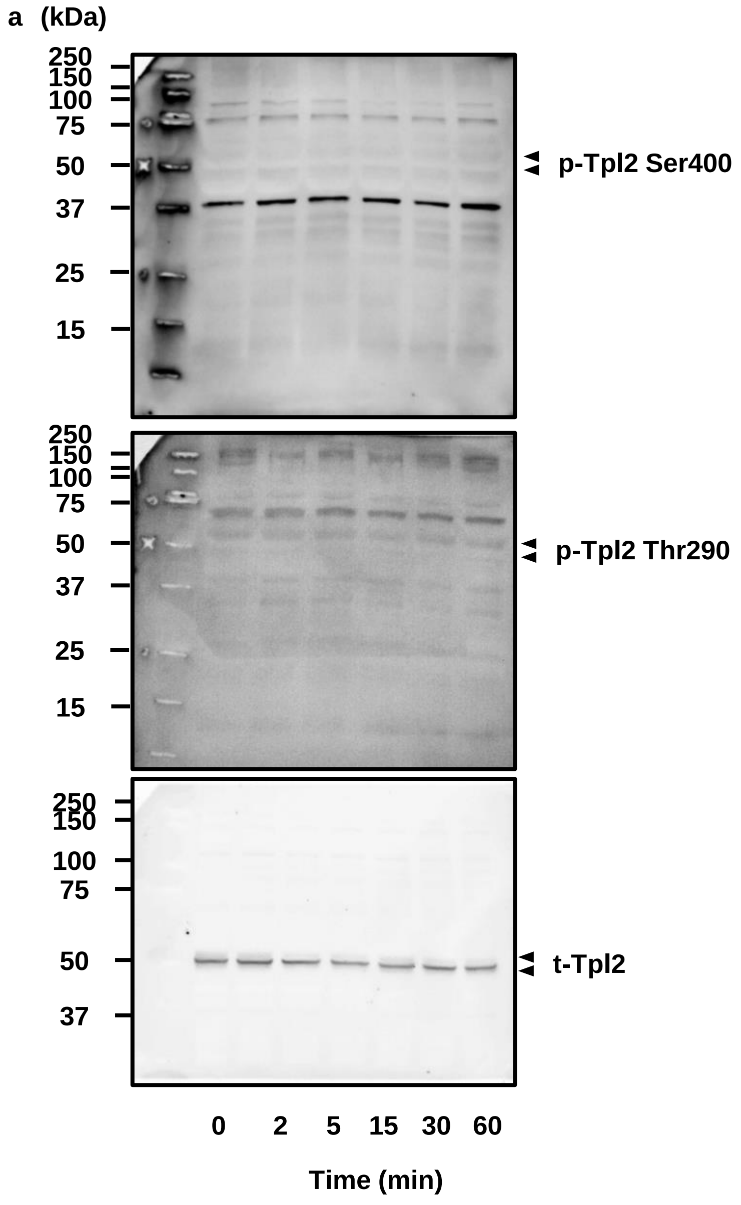

S1 Fig. The effect of IL-1β on the phosphorylation of Tpl2.

When cells were stimulated with 100 pM IL-1β for 0-60 min, the phosphorylation of Tpl2 Ser400 and Thr290 could not be detected.
