# Supplementary material for: Tpl2 contributes to IL-1β-induced IL-8 expression via ERK1/2 activation in canine dermal fibroblasts
Source: PLoS One. 2021 Nov 4;16(11):e0259489. doi: 10.1371/journal.pone.0259489 (PMC8568182; doi:10.1371/journal.pone.0259489)
Supplement: S2 Fig — (a) When cells were stimulated with 100 pM IL-1β for 0–60 min, the phosphorylation of MEK was observed. (b) Canine dermal fibroblasts were pretreated with or without the MEK inhibitor U0126 (10 μM) for 1 h and subsequently stimulated with or without IL-1β (100 pM) for 6 h. After stimulation, IL-8 mRNA expression levels were determined. TBP was used as an internal standard. Results have been represented as mean ± SE from biological triplicates. *P < 0.05. (PDF) [file pone.0259489.s002.pdf]

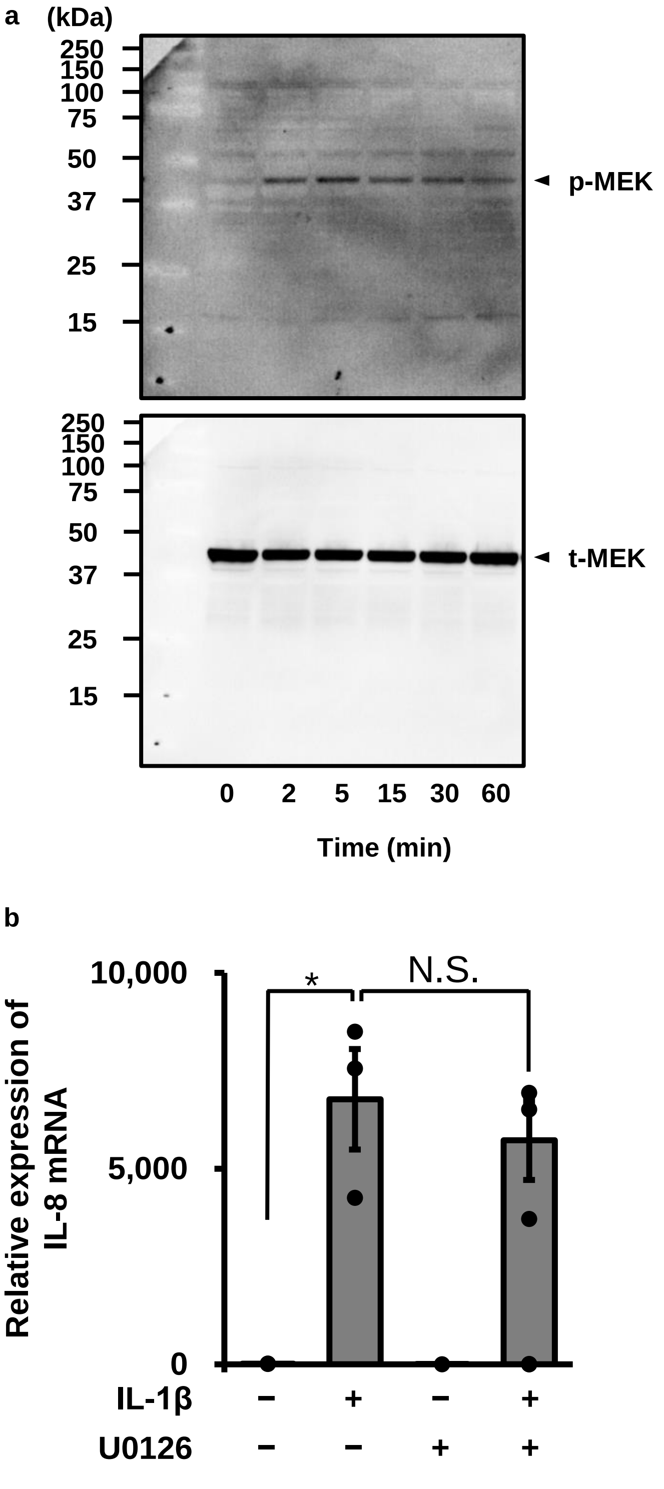

S2 Fig. The contribution of MEK activation to IL-1β-induced IL-8 mRNA expression.

(a) When cells were stimulated with 100 pM IL-1β for 0-60 min, the phosphorylation of MEK was observed.

(b) Canine dermal fibroblasts were pretreated with or without the MEK inhibitor U0126 (10 μM) for 1 h and subsequently stimulated with or without IL-1β (100 pM) for 6 h. After stimulation, IL-8 mRNA expression levels were determined. TBP was used as an internal standard. Results have been represented as mean ± SE from biological triplicates. *P < 0.05.
