# Supplementary material for: Tpl2 contributes to IL-1β-induced IL-8 expression via ERK1/2 activation in canine dermal fibroblasts
Source: PLoS One. 2021 Nov 4;16(11):e0259489. doi: 10.1371/journal.pone.0259489 (PMC8568182; doi:10.1371/journal.pone.0259489)
Supplement: S1 Raw images — (PDF) [file pone.0259489.s003.pdf]

Fig. 3a

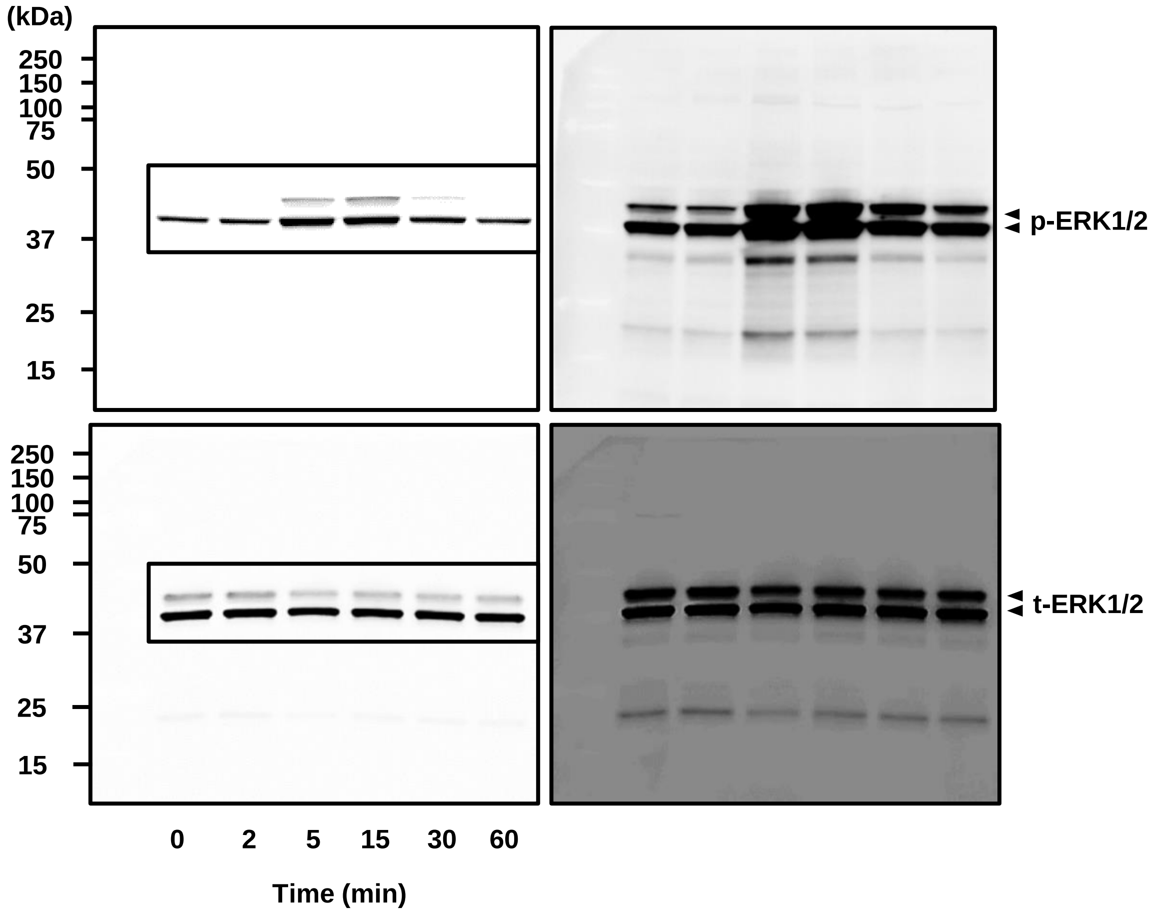

Fig. 3c

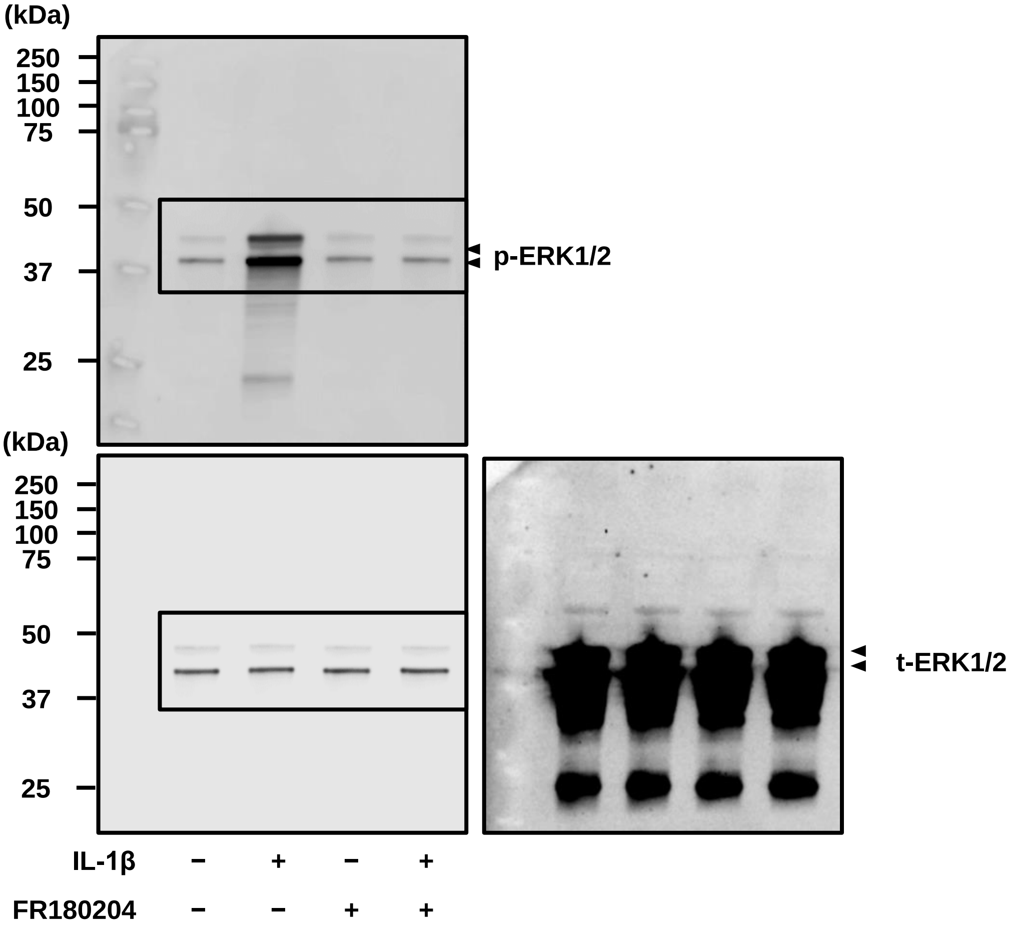

Uncropped images for the blots shown in Fig. 3a and c.

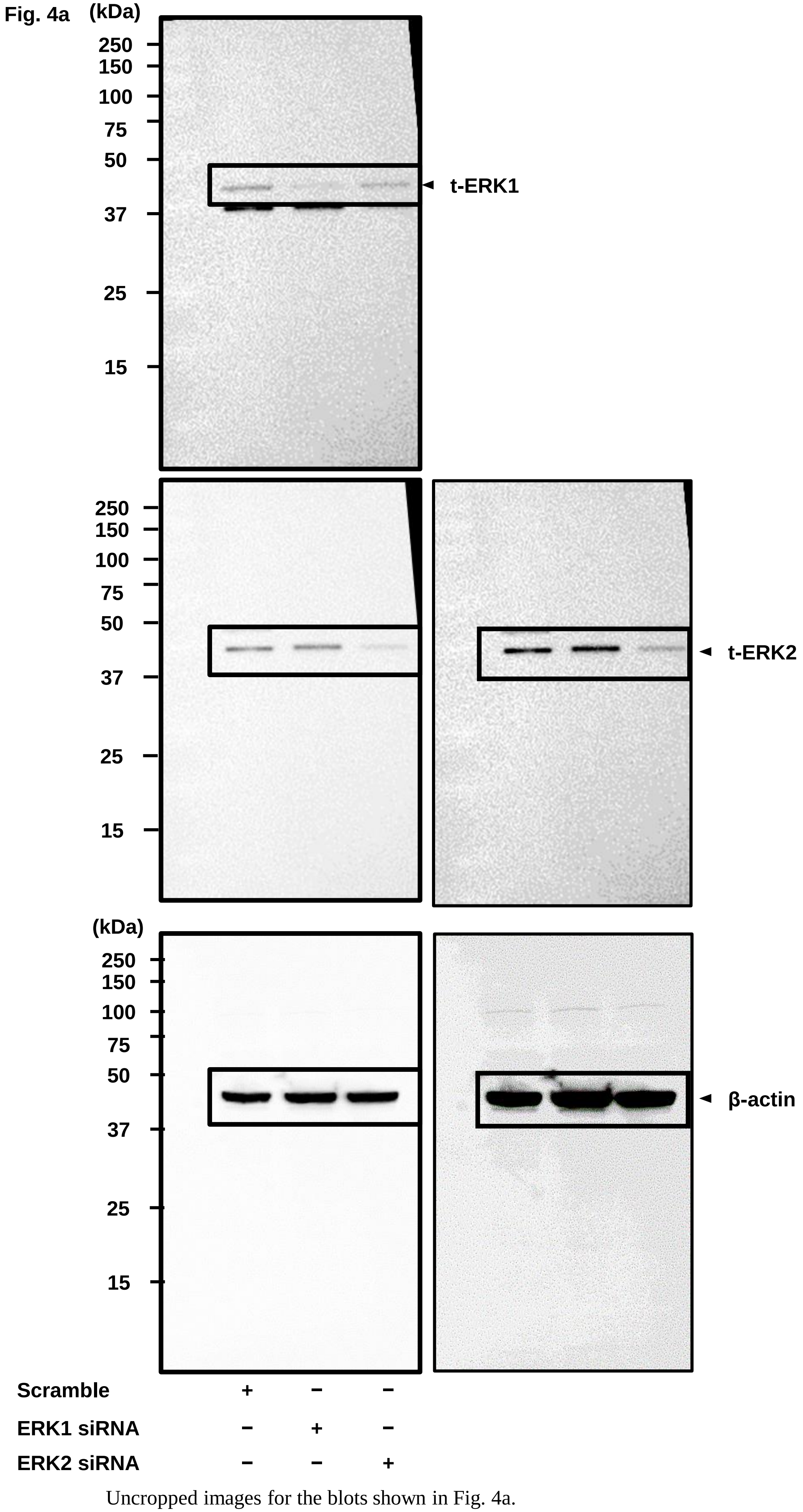

Uncropped images for the blots shown in Fig. 4a.

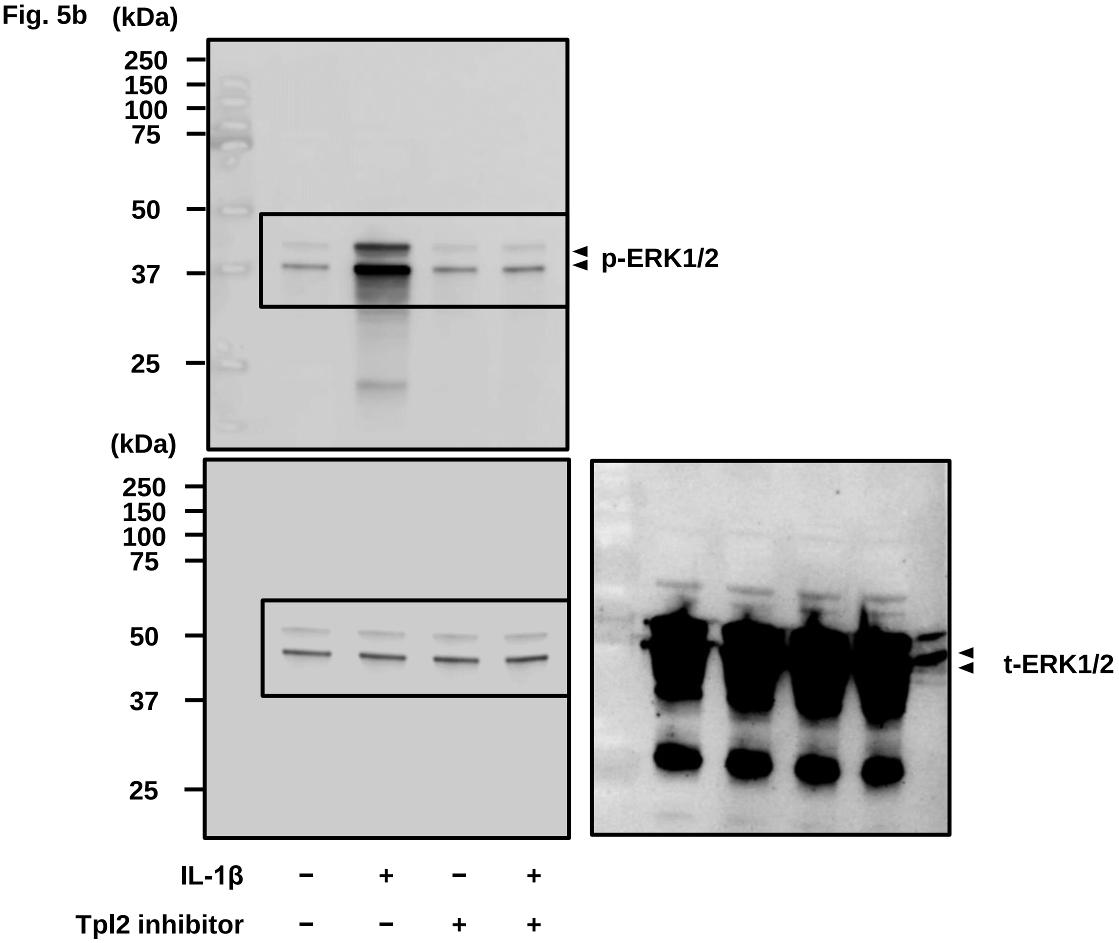

Uncropped images for the blots shown in Fig. 5b.

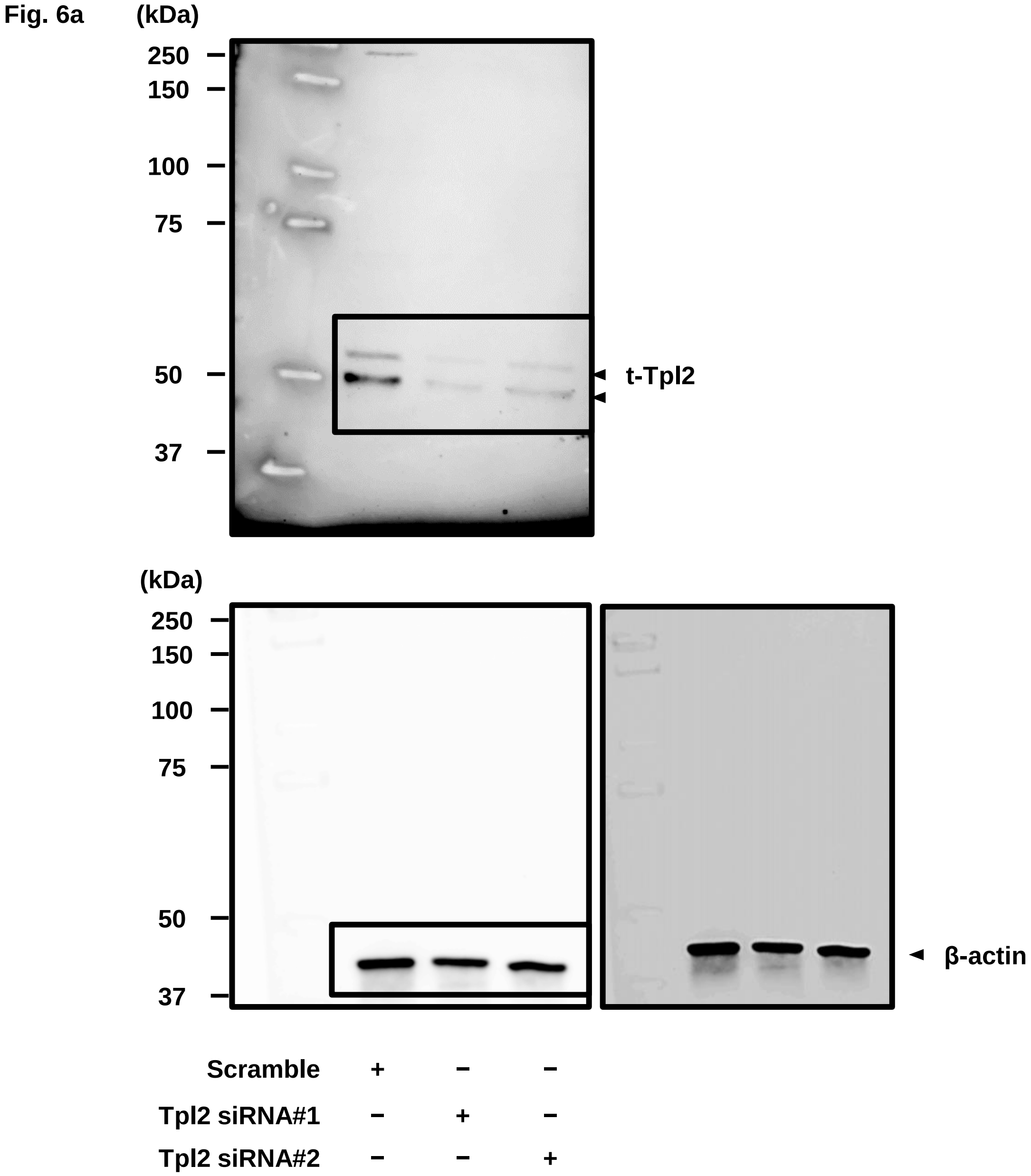

Uncropped images for the blots shown in Fig. 6a.

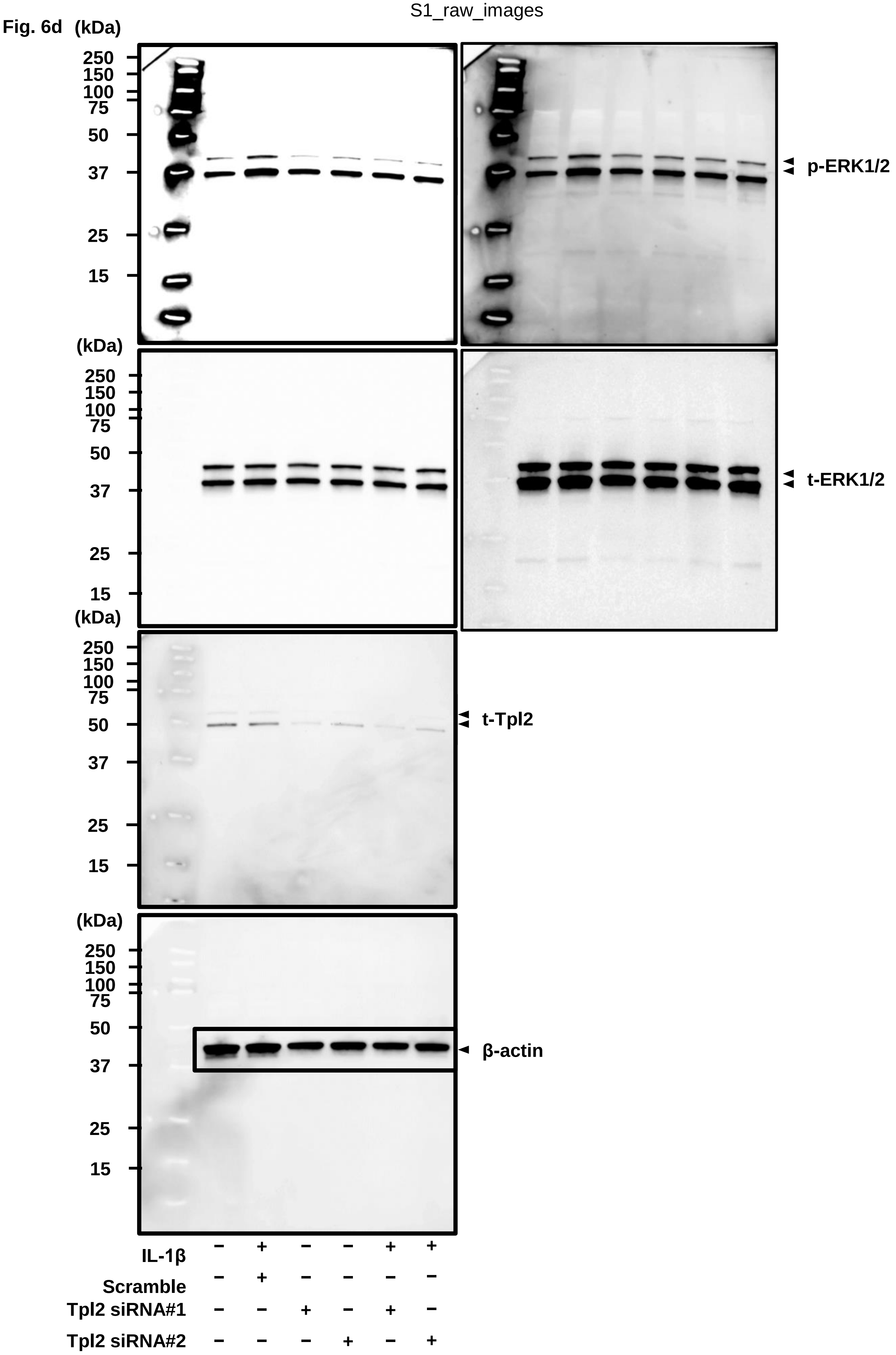

Uncropped images for the blots shown in Fig. 6d..
